# Comparative analysis of crop rotation systems: the impact of ginger (*Zingiber officinale*) and sponge gourd (*Luffa aegyptiaca*) residues on growth of Chinese cabbage (*Brassica rapa* var. *chinensis*)

**DOI:** 10.3389/fpls.2024.1428943

**Published:** 2024-10-11

**Authors:** Zhangliang Yao, Jiashun Miao, Baojun Wang, Weidong Xu, Yeqing Wang, Qiang Lu, Jidong Zhang

**Affiliations:** ^1^ Institute of Eco-Environmental Sciences, Jiaxing Academy of Agricultural Sciences, Jiaxing, China; ^2^ Xianghu Laboratory, Hangzhou, China; ^3^ The Promotion Station of Plant Protection, Fertilizer Utilization and Rural Energy Technology of Jiaxing, Jiaxing, China; ^4^ Chongfu Zhang Jidong family farm, Jiaxing, China

**Keywords:** rotation cropping, soil physicochemical properties, rhizosphere microorganisms, Chinese cabbage, ginger, sponge gourd

## Abstract

Continuous cropping in greenhouse cultivation often leads to increased pest and disease problems, reducing crop quality and yield. Crop rotation is a common strategy to address these issues. This study compared the growth of Chinese cabbage (*Brassica rapa* var. *chinensis*) following rotations with ginger (*Zingiber officinale*) and sponge gourd (*Luffa aegyptiaca*). The Chinese cabbage exhibited normal growth following ginger rotation but showed abnormal growth after sponge gourd rotation. The study investigated the underlying causes by analyzing soil physicochemical properties and rhizosphere microbial communities of Chinese cabbage using 16S rRNA and ITS sequencing. The results revealed that soil from ginger–Chinese cabbage rotation had higher levels of soil organic carbon (SOC) and available phosphorus (AP), but lower total nitrogen (TN) and available potassium (AK). Despite similar alpha-diversity for both bacterial and fungal communities, distinct bacterial and fungal community structures between two rotation cropping systems were observed. This suggests that even if the alpha-diversity does not change, the composition of the microbial community can shift in ways that might influence soil health and plant growth. Furthermore, redundancy analysis revealed a significant correlation between microbial community structures and soil physicochemical properties of two rotation cropping systems. The SOC and TN were revealed to be the most significant of the investigated soil physicochemical parameters with respect to the variation of both bacterial and fungal assemblages, respectively. The identified biomarkers in bacterial community composition further emphasize the potential for specific microbes to influence crop health positively or negatively. We found that the indicator genera of the bacterial community composition of the ginger–Chinese cabbage rotation system were *Amycolatopsis* (genus), *Pseudonocardiales* (order), *Pseudonocardiaceae* (family), and *Amycolatopsis mediterranei*, which are known as producers of secondary metabolites, such as antibiotics. These findings highlight the importance of crop selection in rotation strategies for optimizing agricultural outcomes.

## Introduction

Continuous cropping obstacles pose a significant challenge in agricultural production, often exacerbating pest and disease problems, and leading to declines in crop quality and yield (Bi et al., 2023; Zhang et al., 2024). In greenhouse cultivation, the challenges posed by continuous cropping obstacles on vegetable production are particularly severe. To overcome continuous cropping obstacles, various strategies may be employed, including soil improvement, alteration of crop types or farming patterns, reasonable fertilizing, application of soil microbial regulators, and cultivation of resistant varieties. Among these strategies, crop rotation is frequently employed by farmers. For example, alternating wet and dry cultivation of chrysanthemum with rice has been shown to substantially decrease disease in chrysanthemum crops. Additionally, rotating legumes with wheat provides both economic and ecological benefits (Rebolledo-Leiva et al., 2022). Rotation cropping has potential to enhance both product quality and quantity, effectively mitigating the pest and disease problems associated with continuous cropping (Kelley et al., 2003; Dayegamiye et al., 2017; Zhao et al., 2020).

Soil microorganisms, particularly those in the rhizosphere, play a pivotal role in the challenges of continuous cropping (Ma et al., 2023). For instance, root rot disease in continuous cropping systems underscores the intricate relationship between crop health and the rhizosphere microbial community (Bi et al., 2023). Studies have demonstrated that continuous cropping can reduce bacterial community richness and diversity, as observed in the muskmelon rhizosphere soil, and shift microbial communities in ways detrimental to plant growth, such as in potatoes (Wang et al., 2023; Zhang et al., 2023; Xing et al., 2024). Selecting crop varieties tolerant to these conditions, such as certain soybean, or introducing beneficial microbes, like *Bacillus cereus* WL08, can mitigate these obstacles through microbial mediation and soil health enhancement (Liu et al., 2023; Zhang et al., 2023). Reintroducing ryegrass to continuous cropping soil can alleviate continuous cropping obstacles in tobacco cultivation by enhancing soil nutrients and the soil microbiome (Zhou et al., 2023).

**Figure 1:**
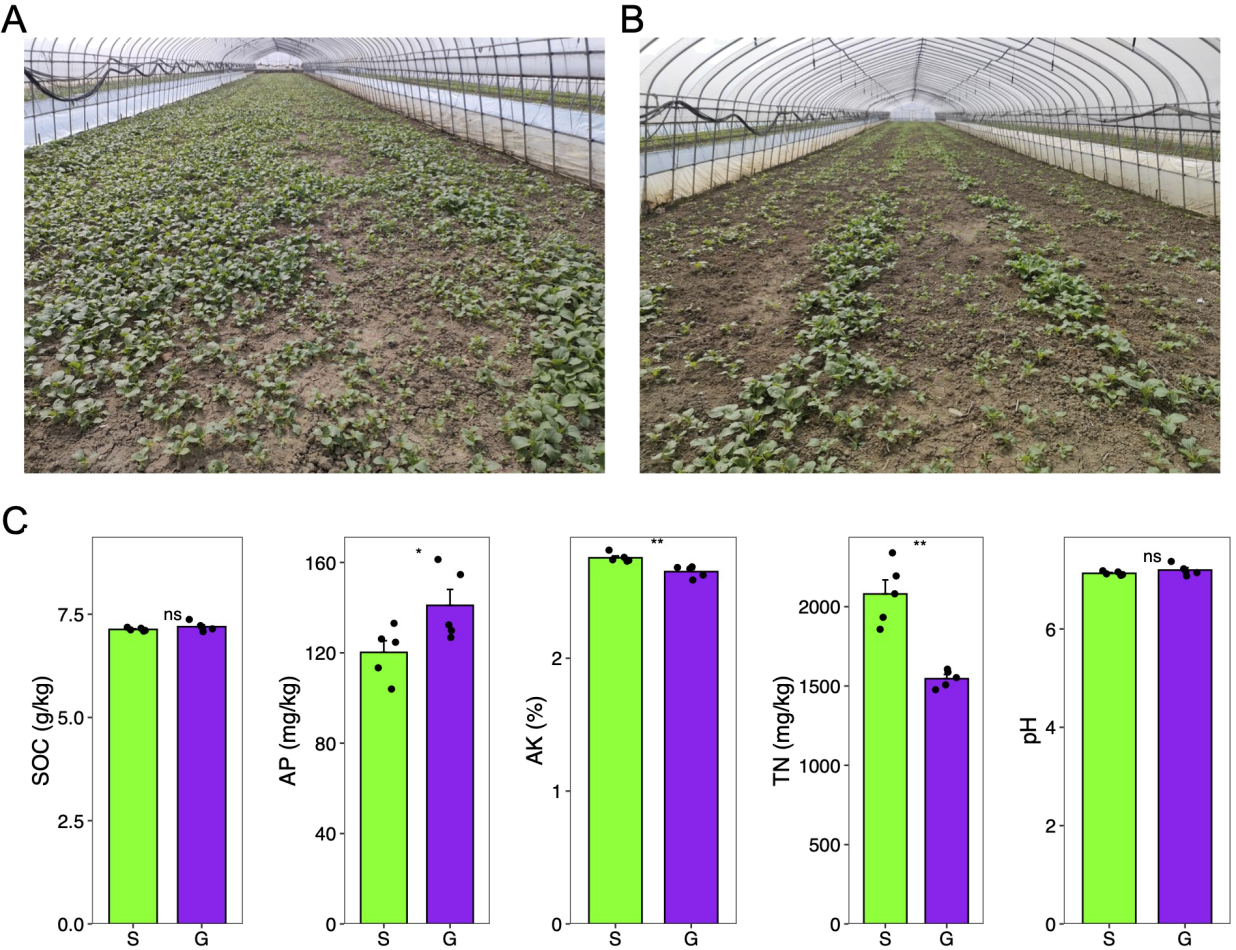
Soil physicochemical property measurement of two rotation cropping systems. **(A)** Photo taken for the Chinese cabbage rotated after ginger in a greenhouse. **(B)** Photo taken for the Chinese cabbage rotated after sponge gourd in a greenhouse. **(C)** Soil physicochemical properties of soil sampled from the ginger–Chinese cabbage (G) rotation cropping system and sponge gourd–Chinese cabbage (S) rotation cropping system after the harvest of Chinese cabbage. Five sampling sites were chosen for both G and S. SOC, soil organic carbon; TN, total nitrogen; AP, available phosphorus; AK, available potassium. Student t test was conducted to compare the difference of these soil physicochemical properties between two groups. ns, non-significant; *significant level of 0.05; **significant level of 0.01.

Several studies have revealed that rotation cropping can overcome continuous cropping obstacles by altering the soil microbial community structure (Wang et al., 2022). Furthermore, long-term crop rotation can influence certain soil nutrients (Neugschwandtner et al., 2022). For example, compared with the continuous tobacco–rice rotation system, continuous tobacco cultivation increases the content of soil soluble organic carbon, total nitrogen, available phosphorus, and available potassium but reduces bacterial diversity and alters community composition (Wang et al., 2022). Moreover, the stage of crop rotation has a more significant impact on the soil microbial community than fertilization (Xie et al., 2022). Moreover, it is important to note that not all crop rotation strategies produce beneficial outcomes for plant growth and disease resistance. Our research presents a counterexample in which a rotation system fails to achieve these positive effects. After the autumn harvest of greenhouse crops such as sponge gourd, eggplant, ginger, corn, and melons, Chinese cabbage is typically sown as the subsequent crop in the rotation in the middle and lower reaches of Yangtze River, China. Over extended periods of agricultural practice, we have observed that Chinese cabbage following a sponge gourd rotation exhibits less vigor compared with when it follows ginger (**Figures 1A, B**). However, the impact of different preceding crops on the rhizosphere microbiota and soil nutrients of Chinese cabbage as a subsequent crop remains largely unexplored. To address this gap, we conducted an experiment to examine how preceding crops of sponge gourd and ginger influence the rhizosphere microbiota and soil nutrient content of Chinese cabbage as the subsequent crop.

## Materials and methods

### Site description

The experiment was conducted in two greenhouses at Liuliang Village, Chongfu Town, Tongxiang City, Jiaxing, Zhejiang Province (latitude and longitude: 30.5247, 120.4831). The two greenhouses had similar conditions. Based on the typical planting practices of these crops in Zhejiang Province, sponge gourd was planted on 04/03/2023, and ginger was planted on 21/02/2023, each in separated greenhouses. Both crops were managed conventionally. Following the harvest of these two crops, the soil in both greenhouses was plowed and loosened for planting Chinese cabbage. Chinese cabbage (De Gao Melbourne hybrid variety, Shandong De Gao Seed Co., Ltd.) was subsequently planted on 21/09/2023, in both greenhouses. The planting method was direct sowing, and cultivation measures such as irrigation and fertilization followed local practices, with the other managements kept consistent between two greenhouses.

### Soil sampling and physicochemical property measurement

After the harvest of Chinese cabbage on 28/12/2023, soil samples were collected from both greenhouses using a five-point sampling method. Soil samples were collected from a depth of 0 cm–20 cm, and after removing stones and plant residues. The soil samples were then air-dried, ground, and sieved to obtain samples for analysis. Basic physicochemical properties of the soil were determined, including soil pH, soil nutrients (total nitrogen, available phosphorus, total potassium), and soil organic matter content. The specific methods are briefly described as follows: Soil pH was measured by taking 10 g of soil sample in a beaker, adding 25 ml of distilled water to remove carbon dioxide, sealing the container with a cling film, shaking for 2 min on a horizontal shaker, and then measuring the pH with a pH meter after 30 min of standing. The determination of total nitrogen in soil was based on the Kjeldahl method (Bremner, 1960), which involves converting all nitrogen in the soil into ammonium nitrogen through oxidation–reduction reaction, followed by distillation of ammonia after digestion, absorption of ammonia by boric acid, and titration with standard hydrochloric acid to calculate the total nitrogen content in the soil. Available phosphorus in soil was determined by extracting soil with sodium bicarbonate solution, followed by determination of available phosphorus content using the molybdenum antimony scandium colorimetric method (Song et al., 2019). Total potassium in soil was determined by taking 0.1 g of soil sample, preparing soil digestion solution, ensuring that all potassium elements in the soil are present as potassium ions, diluting the solution, and measuring the potassium ion concentration using atomic absorption spectrophotometry to calculate the total potassium content in the soil (Nielsen, 2010). The determination of soil organic matter mainly involved the oxidation of organic matter in the soil under heating conditions using excess potassium dichromate–sulfuric acid solution, titration of excess potassium dichromate with standard ferrous sulfate solution, and calculation of soil organic matter content (Roper et al., 2019).

### Sampling for rhizosphere microbiome analysis

On 18/10/2023, after the harvest of Chinese cabbage, soil samples were collected from both types of greenhouses using a five-point sampling method. In the sponge gourd–Chinese cabbage rotation cropping greenhouse, sampling was conducted avoiding the original planting furrows of sponge gourd. The roots of Chinese cabbage were excavated with a shovel to a depth of 10 cm, and soil adhering to the roots was removed by shaking, followed by cutting off the roots with scissors.

### DNA extraction and Illumina sequencing

The genomic DNA was extracted from 0.5 g of Chinese cabbage root using the CTAB mothed. The purity of DNA was checked by a NanoDrop spectrophotometer (Thermo Fisher Scientific, United States). The V3–V4 regions of 16S rRNA were amplified with the primers 338F (ACTCCTACGGGAGGCAGCA) and 806R (GGACTACHVGGGTWTCTAAT) to demonstrate the composition and structure of the bacterial community. The ITS (internal transcribed spacer) regions of soil samples were amplified with a forward primer (CTTGGTCATTTAGAGGAAGTAA) and a reverse primer (GCTGCGTTCTTCATCGATGC) to demonstrate the composition and structure of the fungus community. After purification and library construction, the amplicons were sequenced on an Illumina HiSeq platform (Illumina, San Diego, United States).

### Microbial data analysis

The raw reads of Illumina sequencing were firstly filtered by using Trimmomatic (v0.33) (Bolger et al., 2014). Then, the primer sequences were identified and removed by using cutadapt (version 1.9.1) (Martin, 2011). The filtered reads were denoised into ASVs by dada2 in QIIME2 2020.6 (Bolyen et al., 2019). ASVs were referred to as a feature in following text. Taxonomy annotation was performed based on the features. Microbial community composition at phylum, class, order, family, genus, and species levels was revealed according to taxonomy annotation (Yang et al., 2023). The alpha diversity indexes were calculated using QIIME2 to assess the microbial abundance and diversity, and the differences between two rotation cropping groups were tested by Student t-test. Microbial beta-diversity was quantified with two axes of a non-metric multidimensional scaling (NMDS) analysis of Jaccard and Bray–Curtis dissimilarities in the OTU community matrix using the “vegan” package (version 2.6-4). PERMANOVA (permutational multivariate analysis of variance) for comparing the community structure between two rotation cropping systems was analyzed with adonis function within the vegan package, and the Bray–Curtis method was employed to calculate the sample distance. ANOSIM (analysis of similarities) for two rotation cropping systems was conducted using the anosim() function within the “vegan” package, and the Bray–Curtis method was employed to calculate the sample distance. ANOVA (analysis of variance) was performed on taxa relative abundance to identify biomarkers with a statistically significant difference between two rotation cropping groups. To obtain the biomarkers of microbial taxa between two rotation cropping systems, we performed LEfSe (line discriminant analysis effect size) analysis by using the microeco package (version 1.6.0) (Segata et al., 2011). Non-parametric factorial Kruskal–Wallis (KW) sum-rank test was firstly used to detect features with significant differential abundance and then taxa with significant differential abundance. Linear discriminant analysis (LDA) was then used to estimate the effective size of each differentially abundant feature between two rotation cropping systems. Taxa with an adjusted P value below 0.05 and an LDA score above 3 will be kept as candidate biomarkers.

## Results

### Growth performance and soil physicochemical characteristics of two rotation cropping systems

Over an extended period of agricultural practice, it was observed that Chinese cabbage (*Brassica rapa* var. *chinensis*) following sponge gourd rotation exhibited less vigor compared with that following ginger rotation (**Figures 1A, B**). To elucidate this disparity and potentially inform agricultural practices and soil management strategies, an initial analysis was conducted on soil physicochemical parameters, including pH, soil organic carbon (SOC), total nitrogen (TN), available phosphorus (AP), and available potassium (AK), given that soil physicochemical properties are critical determinants of agricultural productivity and ecological sustainability. Parameters such as pH, SOC, TN, AP, and AK serve as essential indicators of soil health, each contributing significantly to plant growth and soil ecosystem functionality (Iqbal et al., 2021; Hartmann and Six, 2023; Philippot et al., 2024). Our findings here indicate that the levels of SOC and AP in the soil sampled after the harvest of Chinese cabbage from the ginger–Chinese cabbage (G) rotation were significantly higher than those in the sponge gourd–Chinese cabbage (S) rotation (**Figure 1C**; **Table 1**). Conversely, the concentrations of soil TN and AK were significantly lower in the ginger–Chinese cabbage (G) rotation compared with the sponge gourd–Chinese cabbage (S) rotation (**Figure 1C**; **Table 1**). The soil pH showed no significant difference between the two rotation cropping systems. The SOC showed a significant negative correlation with AK (Pearson r = −0.79, p < 0.01), and TN (Pearson r = −071, p < 0.05). Additionally, TN showed a significant negative correlation with AP (Pearson r = −0.68, p < 0.05) and a positive correlation with AK (Pearson r = 0.68, p < 0.05).

**Table 1:** Soil physicochemical properties of different cropping systems.

Sample ID	pH	SOC (g/kg)	TN (mg/kg)	AP (mg/kg)	AK (%)
**S1**	7.18	23.1	1,850	113	2.81
**S2**	7.16	24.7	2,340	104	2.76
**S3**	7.12	21.2	1,930	126	2.74
**S4**	7.09	25.1	2,080	133	2.73
**S5**	7.11	23.4	2,200	125	2.74
**G1**	7.37	28.8	1,470	161	2.68
**G2**	7.17	33.2	1,610	127	2.69
**G3**	7.15	27.9	1,560	155	2.63
**G4**	7.08	36.3	1,590	130	2.59
**G5**	7.22	35.2	1,500	132	2.67

### Comparison of alpha-diversity of the rhizosphere microbial community for two rotation systems

**Figure 2:**
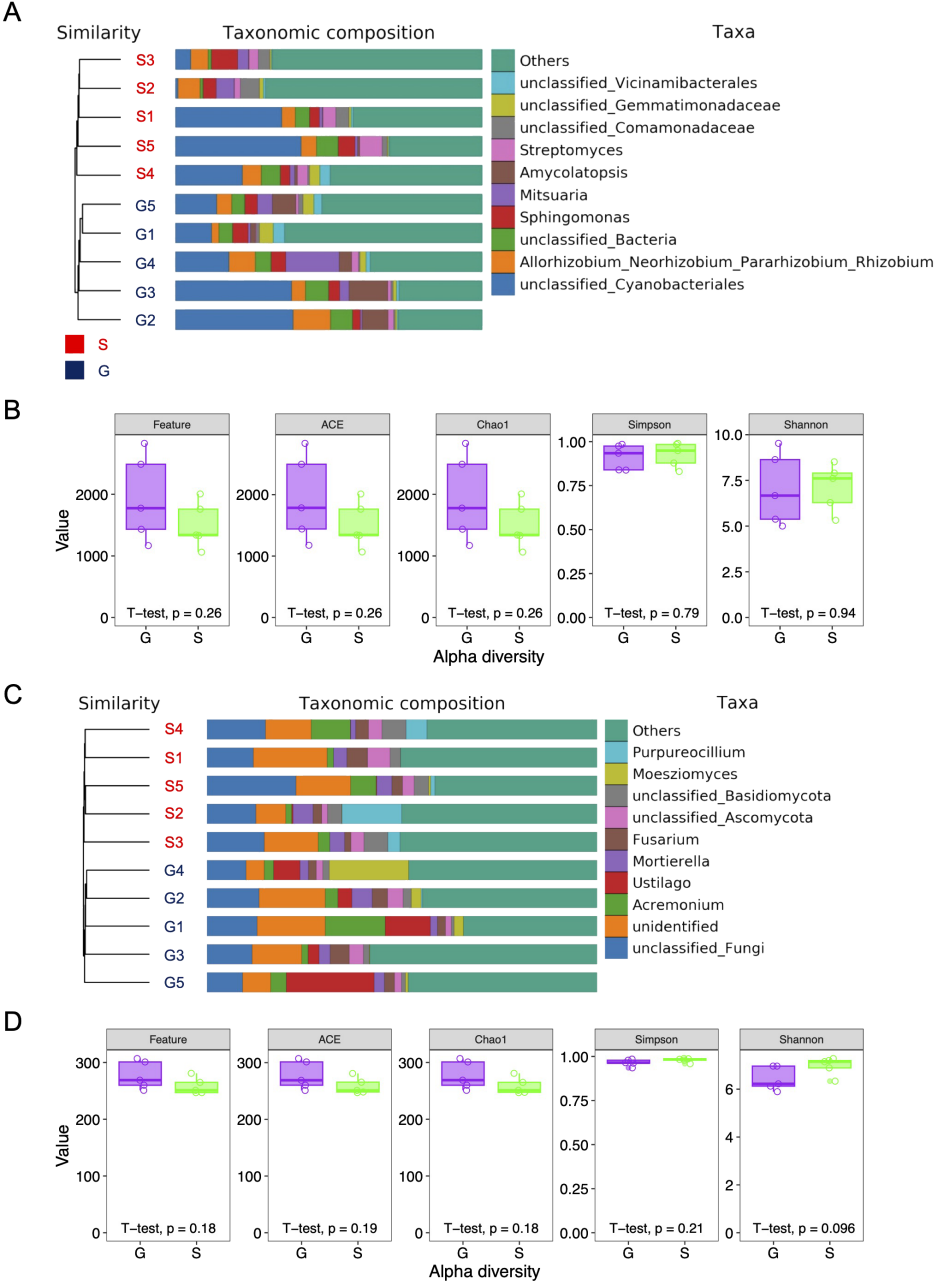
Alpha diversity index of rhizosphere microbial community of Chinese cabbage from two rotation cropping systems. **(A)** Bar plot of the taxonomic distribution of 16S rRNA sequences at the phylum level. The area of each block indicates abundance of phylum in the corresponding sample. The samples were clustered by using UPGMA with binary Jaccard distance. G, ginger–Chinese cabbage rotation cropping system; S, sponge gourd–Chinese cabbage rotation cropping system. **(B)** Alpha diversity indices, including Feature, ACE, Chao1, Simpson, and Shannon, were calculated based on the 16S rRNA sequencing data. Five sites were chosen for two rotation systems (G and S), respectively. **(C)** Bar plot of the taxonomic distribution of ITS sequences at the phylum level. The samples were clustered by using UPGMA with binary Jaccard distance. **(D)** Alpha diversity indices, including Feature, ACE, Chao1, Simpson, and Shannon, were calculated based on the ITS sequencing data. Five sites were chosen for two rotation cropping systems (G and S), respectively.

To explore the differences in alpha-diversity within the rhizosphere microbial communities between the two rotation cropping systems, both 16S rRNA and ITS sequencing were conducted on identical samples. In the 16S DNA sequencing, a total of 800,071 paired-end reads were obtained across 10 samples, with 751,194 clean reads derived following quality control and assembly of paired-end (PE) reads (**Supplementary Table 1**). A minimum of 74,578 and an average of 75,119 clean reads were generated per sample. In ITS sequencing, 800,040 paired-end reads were generated from 10 samples, with 373,084 clean reads obtained following quality control and assembly of PE reads. A minimum of 23,903 and an average of 37,308 clean reads were generated per sample (**Supplementary Table 2**). Alpha-diversity, indicative of species richness and evenness within a community, was assessed using indices including Chao1, Simpson, and Shannon. As illustrated in **Figure 2**, for both 16S and ITS sequenced samples, the mean values of Feature, ACE, and Chao1 for the ginger–Chinese cabbage rotation cropping system (G) were higher than those for the sponge gourd–Chinese cabbage rotation cropping system (S). However, these differences in alpha-diversity, pertaining to both bacterial and fungal communities, were not statistically significant between the G and S systems (**Figure 2A**; **Supplementary Tables 3**, **4**). Notably, the S system exhibited higher mean Simpson and Shannon values compared with the G system, yet these differences were also not statistically significant. Furthermore, the count of operational taxonomic units (OTUs) indicated no significant differences between the G and S systems (**Supplementary Tables 5**, **6**). In conclusion, there is no significant difference in the alpha-diversity of the rhizosphere microbial community between the two rotation cropping systems.

### Influence of rotation cropping systems on rhizosphere bacterial and fungal community structure

**Figure 3:**
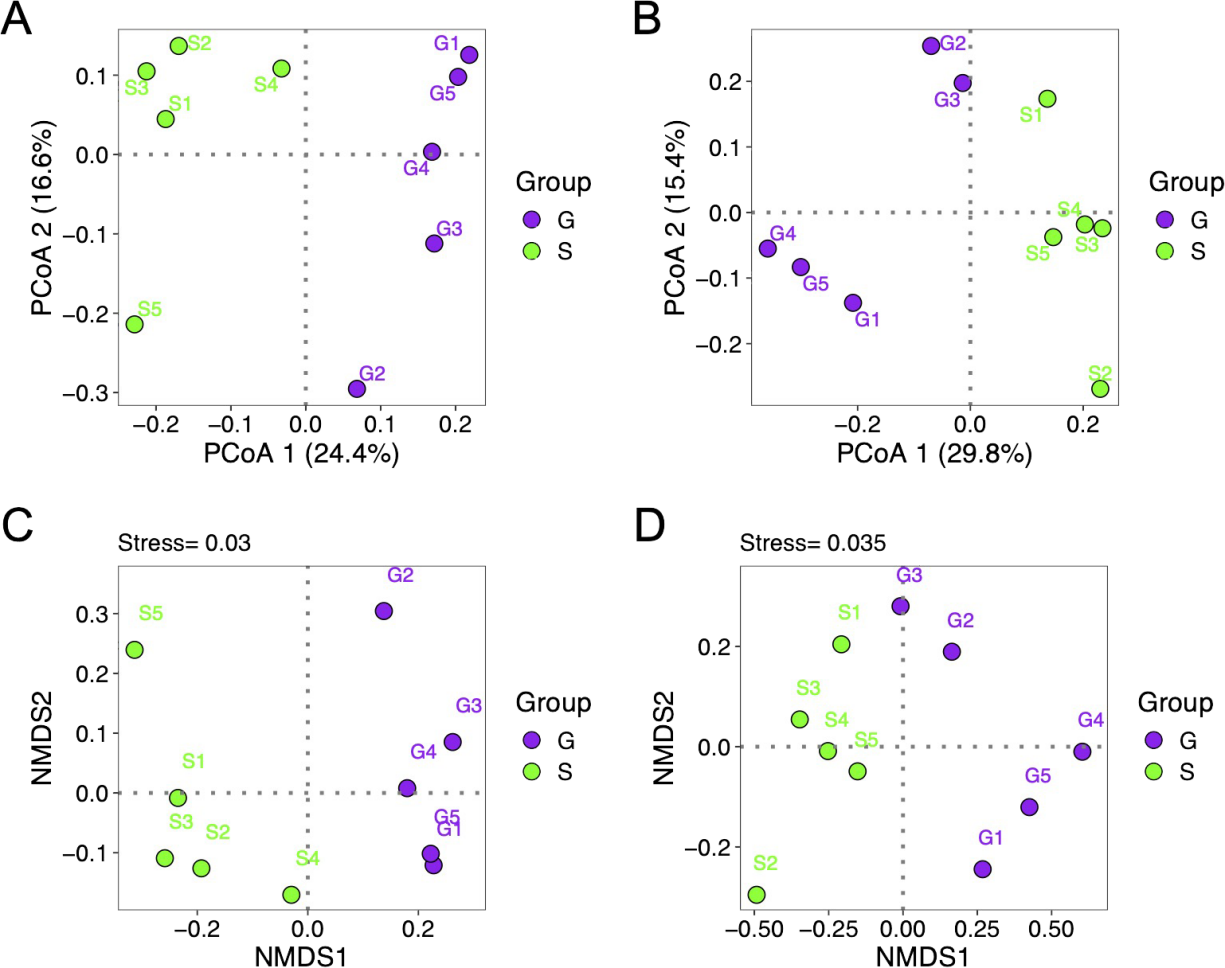
Beta-diversity index of rhizosphere microbial community of Chinese cabbage from two rotation cropping systems. **(A)** Principal coordinate analysis (PCoA) of rhizosphere bacterial communities of Chinese cabbage from two rotation cropping systems (based on the binary Jaccard distance). Each dot represents a sample. Purple and green colors were used to indicate two groups S and G. G is short for the ginger–Chinese cabbage rotation cropping system; S is short for the sponge gourd–Chinese cabbage rotation cropping system. **(B)** PCoA of rhizosphere fungal communities of Chinese cabbage (based on the Bray–Curtis distance). **(C)** Non-metric multidimensional scaling (NMDS) plot of rhizosphere bacterial communities of Chinese cabbage from two rotation cropping system using binary Jaccard distance. Stress value below 0.05 indicates good fit. **(D)** NMDS plot of rhizosphere fungal communities of Chinese cabbage using Bray–Curtis distance.

This study employed both principal coordinate analysis (PCoA) and non-metric multidimensional scaling (NMDS) analysis to investigate the differences of rhizosphere microbial community composition between the two rotation cropping systems. The PCoA using binary Jaccard distance on 16S rRNA sequences revealed a distinct separation of rhizosphere fungal communities of Chinese cabbage along the PCoA1 axis, accounting for 29.8% of the variance (**Figure 3A**). Similarly, PCoA using Bray–Curtis distance on ITS sequences demonstrated a clear separation of rhizosphere bacterial community composition between the two rotation cropping systems along the PCoA1 axis, explaining 24.4% of the variance (**Figure 3B**). NMDS based on binary Jaccard distance depicted two distinct groupings of rhizosphere samples based on the cropping system employed for both 16S rRNA and ITS sequencing data (**Figures 3C, D**). The stress values for NMDS of 16S rRNA and ITS data were 0.03 and 0.035, respectively. Furthermore, PERMANOVA (permutational multivariate analysis of variance) test based on Bray–Curtis distance showed that both the rhizosphere bacterial (R^2^ = 0.2060, p = 0.043) and fungal (R^2^ = 0.2554, p = 0.01) communities of Chinese cabbage of two rotation cropping systems were significantly different. Moreover, MRPP (multi-response permutation procedure and mean dissimilarity matrix) analysis further confirmed significant differences of bacterial (p = 0.049) and fungal (p = 0.009) community structures between two rotation cropping systems. Taken together, the composition of the rhizosphere microbial community exhibited significant change with different preceding crops (ginger and sponge gourd), which underscore the significant impact of rotation cropping practice on the structure of rhizosphere bacterial and fungal communities.

**Figure 4:**
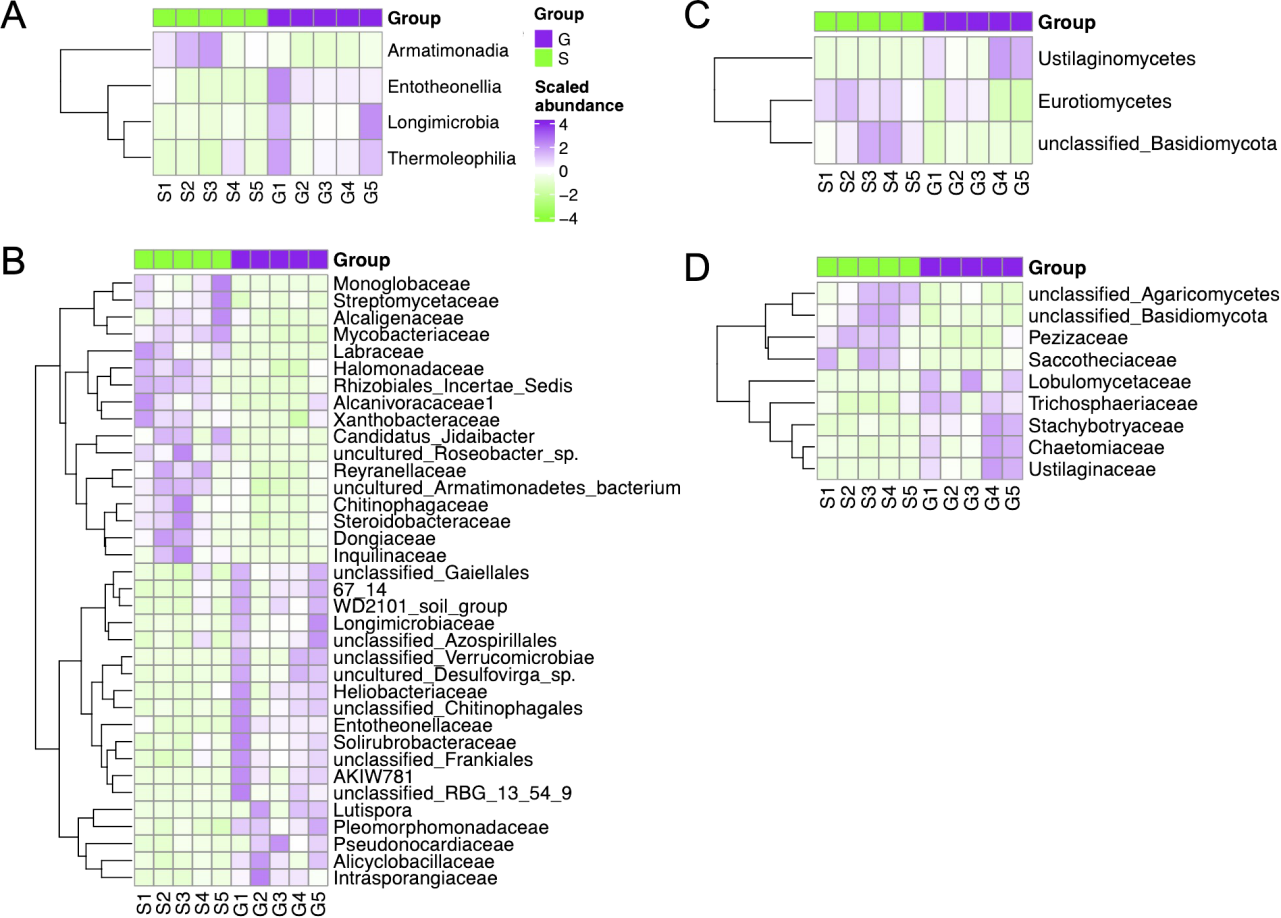
Heatmap of bacterial and fungal composition with significant relative abundance difference between the two rotation cropping systems by ANOVA. **(A)** Analysis of variance (ANOVA) for bacterial composition (class level) showed significant differences in relative abundance between the two rotation systems. G and S are short for ginger-Chinese cabbage rotation cropping system and sponge gourd–Chinese cabbage rotation cropping system. The heatmap shows the relative abundance scaled across all 10 samples. **(B)** Analysis of variance (ANOVA) for bacterial composition (family level) showed significant differences in relative abundance between the two rotation systems. **(C)** Analysis of variance (ANOVA) for fungal composition (class level) showed significant differences in relative abundance between the two rotation systems. **(D)** Analysis of variance (ANOVA) for fungal composition (family level) showed significant differences in relative abundance between the two rotation systems.

Specifically, the difference of rhizosphere bacterial community composition between ginger–Chinese cabbage (G) and sponge gourd–Chinese cabbage (S) rotation cropping systems is depicted in **Figure 4**. At the class level, Alphaproteobacteria, Armatimonadia, Coriobacteriia, Entotheonellia, Longimicrobia, and Thermoleophilia were the dominant class. Among these, Thermoleophilia, Longimicrobia, and Entotheonellia showed the significantly higher relative abundances in the G rotation cropping system than that in the S rotation cropping system (ANOVA test, p < 0.05). However, the relative abundance of Armatimonadia, Alphaproteobacteria, and Coriobacteriia decreased significantly in G compared with that observed in S (ANOVA test, p < 0.05) (**Figure 4A**). At the family level, 36 families showed a significant difference in relative abundances by ANOVA. Among them, Pseudonocardiaceae was the most dominant family, but their average relative abundance in both G and S rotation cropping systems was very small (**Figure 4B**).

The difference of fungal community composition in ginger–Chinese cabbage (G) and sponge gourd–Chinese cabbage (S) rotation cropping systems is also depicted (**Figure 4**). At the class level, Ustilaginomycetes, Eurotiomycetes, and unclassified_Basidiomycota were the most dominant class. Among them, Ustilaginomycetes showed significant higher relative abundance in the G rotation cropping system compared with that in the S rotation cropping system (ANOVA test, p < 0.05) (**Figure 4C**). At the family level, Ustilaginaceae, Chaetomiaceae, Stachybotryaceae, *Trichosphaeriaceae*, and *Lobulomycetaceae* showed significant higher relative abundance in G compared with that in S (ANOVA test, p value < 0.05) (**Figure 4D**). While the family of unclassified_Basidiomycota, unclassified_Agaricomycetes, Pezizaceae, and Saccotheciaceae showed significant higher relative abundance in S compared with G (ANOVA test, p < 0.05) (**Figure 4D**).

### Microbial community associations with soil physicochemical properties

To find the potential correlations between the distribution of microbial assemblages and the soil physicochemical properties of the two rotation cropping systems, redundancy analysis (RDA) was conducted (Yang et al., 2023). A total of 66.01% variance of bacterial community structure could be explained by all five soil physicochemical properties via RDA. Similarly, 63.15% variance of fungal community structure could be explained by all five soil physicochemical properties via RDA. In conclusion, the microbial community has a strong correlation with the soil physicochemical properties of the two rotation cropping systems.

**Figure 5:**
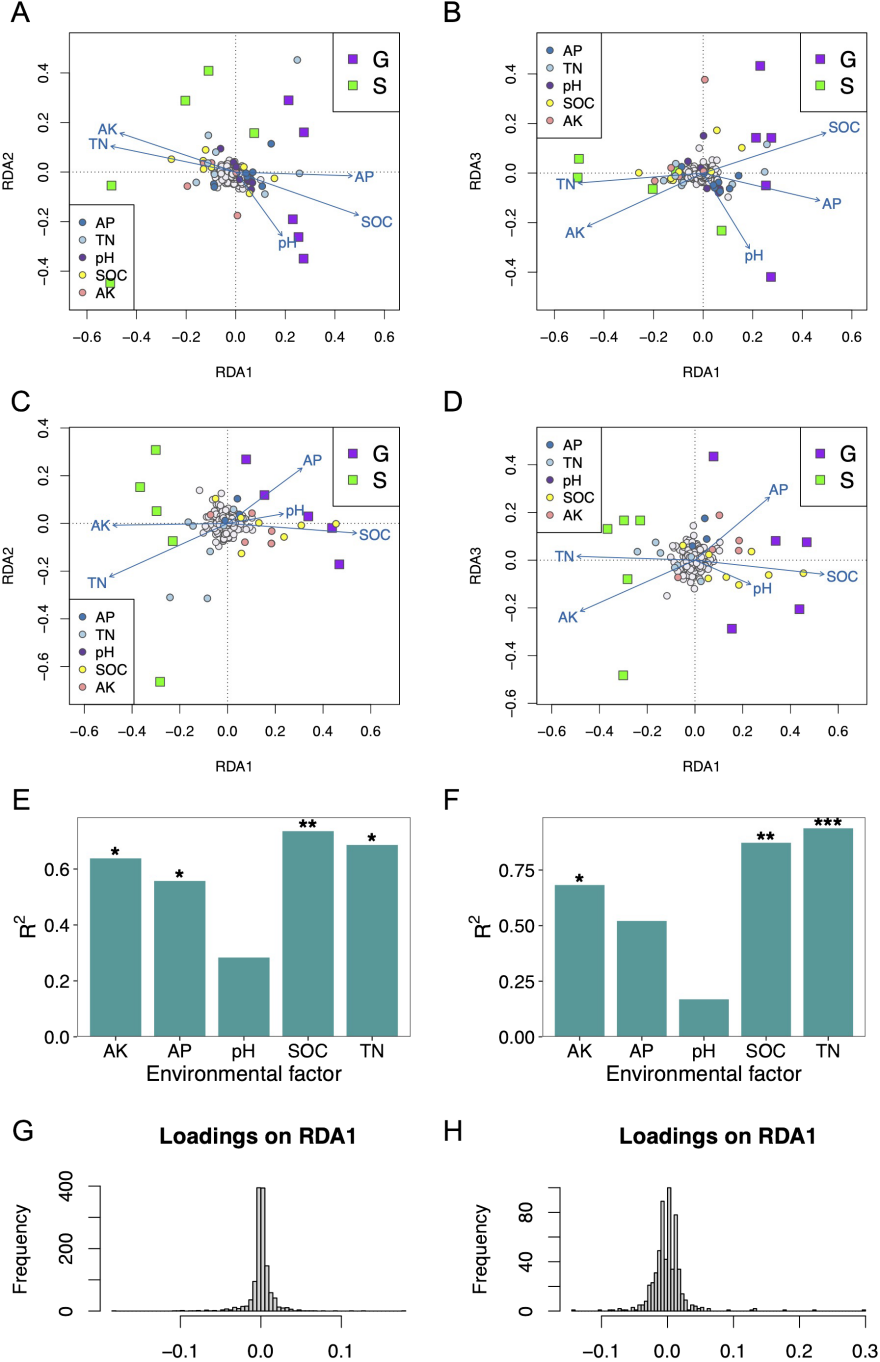
Redundancy analysis (RDA) plots show the correlations between soil physicochemical properties and the microbial community structures of two rotation cropping systems. **(A, B)** were RDA for soil physicochemical properties and bacterial community using the 16S rRNA sequencing data. The taxa (species level) were indicated with circles colored with gray. Taxa significantly correlated with soil physicochemical properties were indicated with correspondence colors shown in the legends. Squares were used to indicate samples collected from rotation cropping systems G and S, respectively. G is short for ginger–Chinese cabbage rotation cropping system, whereas S is short for sponge gourd–Chinese cabbage rotation cropping system. The blue vectors are the soil physicochemical properties. The correlations between the soil physicochemical properties and RDA axes were represented by the length and angle of the arrows. **(C, D)** were RDA for soil physicochemical properties and fungal community using ITS data. **(E)** Effects of soil physicochemical properties on bacterial community structure. **(F)** Effects of soil physicochemical properties on fungal community structure. **(G)** Histogram of bacterial species loadings on constrained axes RDA1. **(H)** The histogram of fungal species loadings on constrained axes RDA1.

From RDA of the bacterial community structure and soil physicochemical properties, we found that these samples from the G group (ginger–Chinese cabbage rotation cropping system) are positively related to high SOC, high AP, low AK, and low TN (**Figures 5A, B**). By contrast, these samples from the S group (sponge gourd–Chinese cabbage rotation cropping) are characterized by high AK level, high TN level, and lower levels of AP and SOC (**Figures 5A, B**). By permutation test (999 replicates), SOC, TN, AK, and AP showed a significant correlation on the bacterial community (**Figure 5E**). On the other hand, the forward selection determined that the bacterial community species gradient was best modeled using only SOC (R^2^ = 0.2561, adjust R^2^ = 0.1631, p = 0.007). Moreover, the adjusted R^2^ of the RDA model only using SOC is close to that using all five soil physicochemical properties (adjusted R^2^ = 0.2351).

From RDA of the fungal community structure and soil physicochemical properties, we found that these samples from the G group are positively related to high SOC, low TN, and low AK (**Figures 5C, D**). By contrast, these samples from the S group are characterized by high AK level, high TN level, and lower levels of SOC (**Figures 5C, D**). By permutation test (999 replicates), SOC, TN, and AK showed a significant correlation on the bacterial community (**Figure 5F**). On the other hand, the forward selection determined that the fungal community species gradient was best modeled using only SOC (R^2^ = 0.2337, adjust R^2^ = 0.1379, p = 0.001). Moreover, the adjusted R^2^ of the RDA model only using SOC is close to that using all five soil physicochemical properties (adjusted R^2^ = 0.1709).

The SOC and TN were revealed to be the most dominant of the investigated soil physicochemical parameters with respect to the variation of both bacterial and fungal assemblages in the two rotation systems, respectively.

Furthermore, we determined candidate species for soil physicochemical environment selection by analyzing the loadings of taxa (species) in the RDA ordination space. Specifically, we extracted these taxa loadings from the three constrained axes, RDA1, RDA2, and RDA3. The histogram of species loadings on constrained axes exhibits a relatively normal distribution (**Figures 5G, H**). Loadings at the center of this distribution do not demonstrate a relationship with environmental predictors. Conversely, taxa with loadings in the tails of the distribution are more likely to be under selection influenced by these soil environmental predictors. These candidate species have already been projected into RDA ordination space in **Figures 5A–D**. For the fungal community, the candidate species that may be under soil physicochemical environment selection include *Purpureocillium lilacinum*, *Xenomyrothecium tongaense*, *Acremonium dichromosporum*, *Podospora bulbillosa*, unclassified *Chaetomiaceae*, unclassified *Agaricomycetes*, *Moesziomyces antarcticus*, *Moesziomyces aphidis*, unclassified *Ustilago*, and unclassified *Basidiomycota* by RDA1 (**Supplementary Table 7**). For the bacterial community, the candidate species that may be under soil physicochemical environment selection include *Amycolatopsis xylanica*, unclassified *Amycolatopsis*, unclassified *Lechevalieria*, unclassified Streptomyces, and other 19 species by RDA1 (**Supplementary Table 8**).

### Biomarker analysis

**Figure 6:**
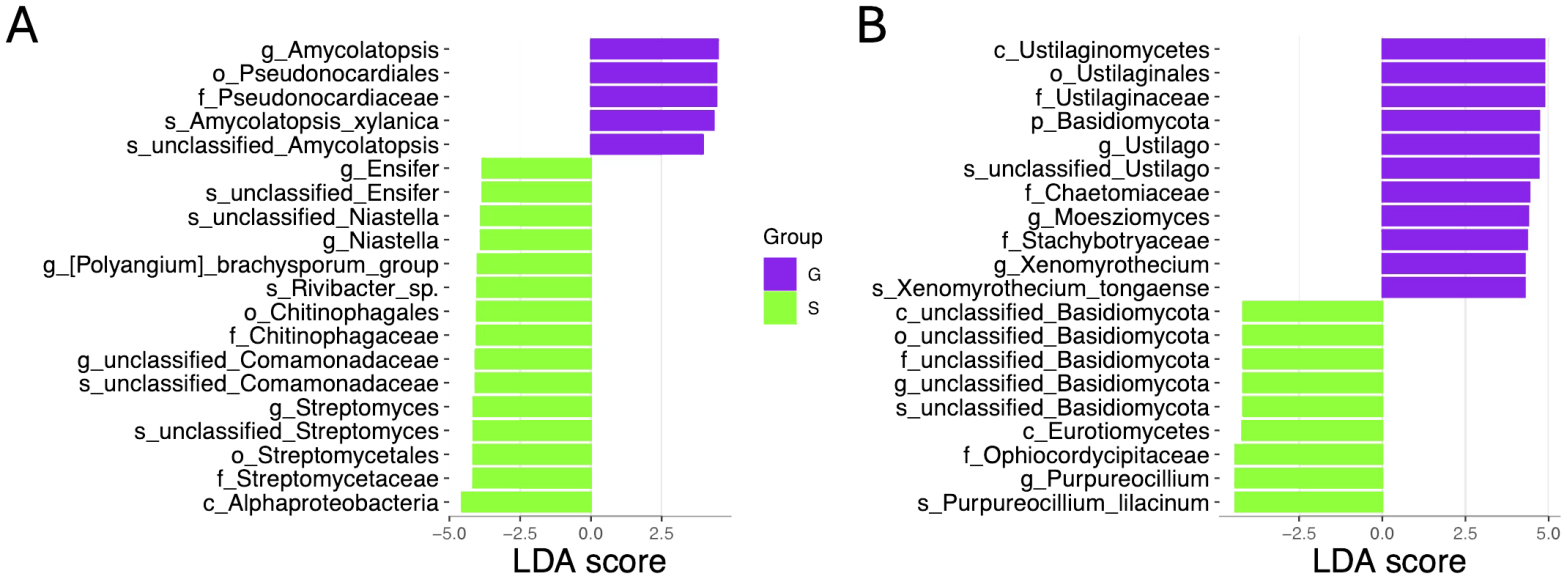
Comparative LEfSe (linear discriminant analysis effect size) results of rhizosphere bacterial and fungal abundance of two rotation cropping systems. **(A)** Identified bacterial biomarkers ranked by the effect size in two rotation cropping systems. Purple and green colors were used to indicate that this taxon is enriched in the sponge gourd–Chinese cabbage (S) rotation cropping system and the ginger–Chinese cabbage (G) rotation cropping system. **(B)** Identified fungal biomarkers ranked by the effect size in different rotation cropping systems.

To identify microbial taxa biomarkers between two rotation cropping systems, we performed LEfSe (line discriminant analysis effect size) analyses. A total of 100 bacterial taxa were identified with significantly distinct relative abundances between the two rotation cropping systems by applying a linear discriminant analysis (LDA) threshold of greater than 3 or less than −3, and an adjusted p-value threshold of less than 0.05. Among the top 20 bacterial biomarkers for the two rotation cropping systems (**Figure 6A**), five taxa, namely, *Amycolatopsis* (genus), *Pseudonocardiales* (order), *Pseudonocardiaceae* (family), *Amycolatopsis xylanica* (species), and an unclassified *Amycolatopsis* species, demonstrated enrichment in the ginger–Chinese cabbage (G) rotation cropping system. Conversely, 15 bacterial taxa, namely, the Alphaproteobacteria (class), Streptomycetaceae (family), Streptomycetales (order), unclassified species of *Streptomyces*, *Streptomyces* (genus), Chitinophagaceae (family), unclassified species within the Comamonadaceae family, unclassified genus within the Comamonadaceae family, Chitinophagales (order), *Rivibacter* sp., *Polyangium brachysporum* group (genus), *Niastella* (genus), unclassified species in the Niastella family, *Ensifer* (genus), and an unclassified species in *Ensifer*, were predominantly found in the sponge gourd–Chinese cabbage (S) rotation cropping system. Furthermore, the analysis identified 51 fungal taxa exhibiting significant differences between the two rotation cropping systems. Among the top 20 fungal biomarkers, for ginger–Chinese cabbage (G) rotation, enriched taxa included classes like Ustilaginomycetes, orders such as Ustilaginales, families like Ustilaginaceae and Chaetomiaceae, genera such as *Ustilago* and *Moesziomyces*, and species including *Xenomyrothecium tongaense* (**Figure 6B**). In contrast, nine fungal taxa were enriched in the sponge gourd–Chinese cabbage (S) system, including unclassified members of the Basidiomycota order, Eurotiomycetes class, *Purpureocillium lilacinum* (species), Ophiocordycipitaceae (family), and the Purpureocillium (genus) (**Figure 6B**). These distinct biomarkers underscore the variation in microbial community structures attributable to different crop rotation practices, potentially reflecting their influence on soil health and crop productivity.

## Discussion

Through analysis of soil physicochemical properties and microbiomes of two different crop rotation systems, we aimed to uncover the reasons behind the significant growth differences observed in the stage of Chinese cabbage. The study focused on soil physicochemical characteristics and rhizosphere microbial diversity, which are key indicators of agricultural productivity and ecological sustainability (Berendsen et al., 2012). The observed differences in soil physicochemical properties between the two rotation cropping systems and their potential impact on the growth of Chinese cabbage offer practical insights for farmers and agronomists. The increase in soil organic carbon (SOC) and available phosphorus (AP) in the ginger rotation system, coupled with the microbial community structure, seems to contribute to the more vigorous growth of Chinese cabbage (**Figure 1**). On the other hand, different growth stages of Chinese cabbage have different requirements for both fertilization types and quantity (Laczi et al., 2017). Furthermore, we only measured the soil physicochemical properties after the harvest of Chinese cabbage. It is a limitation of our experiment. Recently, PSF (plant–soil feedback) provides a solution, which can be used to tell the importance of biota and nutrient on subsequent crop for the rotation system (Koyama et al., 2022).

Plant rhizospheres provide a rich environment where diverse microbial communities, including plant–beneficial microbes and pathogenic microbes, coexist (Berendsen et al., 2012). One intriguing aspect for our study is how the microbial diversity, although not significantly different in alpha-diversity, showed distinct community structures in beta-diversity analysis between the two rotation cropping systems (**Figures 2**, **3**). This also suggests that even if the alpha diversity does not change, the composition of the microbial community can shift in ways that might influence soil health and plant growth. The identified biomarkers in microbial community composition further emphasize the potential for specific microbes to influence crop health positively or negatively (**Figure 4**).

Different plant species could have host-specific microbial communities when grown on the same soil (Xia et al., 2022). The indicator (biomarker) genera of the bacterial community composition of the ginger–Chinese cabbage (G) rotation system were *Amycolatopsis* (genus), Pseudonocardiales (order), Pseudonocardiaceae (family), and *Amycolatopsis mediterranei.* The species within genus *Amycolatopsis* are known as producers of secondary metabolites, such as antibiotics, which are widely used in medicine and agriculture (Kisil et al., 2021). For instance, *Amycolatopsis mediterranei* has been already used for industry-scale production of rifamycin, which plays a vital role in antimycobacterial therapy (Zhao et al., 2010). Similar to *Amycolatopsis*, a number of genera within Pseudonocardiales (order) are also known to produce antibiotics and other bioactive secondary metabolites, some of which have been used in medicine (Franco and Labeda, 2014). Moreover, Pseudonocardiaceae (family) is the only family of order Pseudonocardiales (Franco and Labeda, 2014). For the dominant taxa of the sponge gourd–Chinese cabbage (G) rotation cropping system, these related to antibiotics production taxa also presented, such as unclassified species of *Streptomyces*, *Streptomyces* (genus), Streptomycetales (order), and Streptomycetaceae (family) (Nouioui et al., 2018). The Comamonadaceae family as a dominant taxon in the sponge gourd–Chinese cabbage rotation cropping system, some species within which are pathogens, may be associated with abnormal growth of Chinese cabbage (Willems, 2014).

Although there was no significant difference in the alpha-diversity (species richness and evenness) of rhizosphere microbial communities between the two rotation cropping systems, beta-diversity analysis revealed significant differences in community composition. This suggests that while crop rotation may have a limited impact on the broad-scale diversity of rhizosphere microbial communities, specific rotation systems can significantly alter microbial community structures, potentially affecting soil nutrient cycling and plant health indirectly.

This research adds valuable knowledge to the field of agricultural science, particularly in the context of sustainable farming and crop management strategies. By selecting appropriate crop sequences, it is possible to improve soil conditions, which in turn can lead to healthier and more vigorous plant growth (Koyama et al., 2022). Additionally, plant-soil feedback (PSF) offers a method for choosing crop rotation sequences that optimize agricultural productivity (Koyama et al., 2022). Furthermore, exploring the mechanisms behind the changes in soil nutrients and microbial communities in response to crop rotation could provide deeper insights into sustainable agricultural practices.

## Conclusion

This comparative analysis underscores the significant impact of crop rotation systems on the growth of Chinese cabbage, mediated by changes in soil properties and microbial structure. Our findings offer practical insights for sustainable agricultural practices, emphasizing the strategic selection of rotation crops to improve crop health and yield. This study highlights the importance of selecting appropriate preceding crops for optimizing soil conditions and microbial communities to enhance the growth and health of subsequent crops.

## Data Availability

The data presented in the study are deposited in the figshare repository under the link of https://doi.org/10.6084/m9.figshare.27021463.v2.
